# All-optical binary computation based on inverse design method

**DOI:** 10.1515/nanoph-2021-0467

**Published:** 2021-10-26

**Authors:** Huixin Qi, Zhuochen Du, Jiayu Yang, Xiaoyong Hu, Qihuang Gong

**Affiliations:** State Key Laboratory for Mesoscopic Physics and Department of Physics, Collaborative Innovation Center of Quantum Matter & Frontiers Science Center for Nano-optoelectronics, Beijing Academy of Quantum Information Sciences, Peking University, Beijing 100871, P. R. China; Peking University Yangtze Delta Institute of Optoelectronics, Nantong, Jiangsu 226010, P. R. China; Collaborative Innovation Center of Extreme Optics, Shanxi University, Taiyuan, Shanxi 030006, P. R. China

**Keywords:** all-optical binary computation, half binary adder, inverse design method, shifter

## Abstract

The development of information technology urgently requires ultrafast, ultra-low energy consumption and ultra-high-capacity data computing abilities. Traditional computing method of electronic chips is limited by the bottleneck of Moore’s Law. All-optical computing of photonic chips provides a promising way to realize such high-performance data computing abilities. Until now, it is still a huge challenge to realize all-optical four arithmetic operations at the same time on a photonic chip. Here, we propose a new encoding scheme for all-optical binary computation, including *n*-bit addition, subtraction, multiplication and division. We theoretically present *n*-bit calculation and experimentally demonstrate 1 bit calculation. The computation part includes a half binary adder and a shifter, whose feature sizes are only 2 μm × 19.5 μm and 4 μm × 9 μm, respectively. The half binary adder and shifter consist of three low-loss basic devices through inverse design method. The distance between two adjacent basic devices is smaller than 1.5 μm, within wavelength magnitude scale. The response time is the propagation time of the signal light in a single device, within 100 fs. The threshold energy consumption is within 10 fJ/bit. Our results provide a new method to realize ultrafast, ultra-low energy consumption and ultra-high-capacity data processing abilities all-optical *n*-bit binary computing.

## 1 Introduction

With the rapid development of information technology, the existing computing structures mainly based on electronic processors have been difficult to meet the increasingly urgent requirements of ultra-high-speed, ultra-low energy consumption and large capacity data information processing [1], [2], [3]. A photon has a higher information capacity and speed than an electron. Using photons as the information carriers instead of electrons for information processing provides a possible way to solve the above problems [4], [5], [6]. The arithmetic part is an important component of the central processing unit (CPU), the concept of all-optical calculation provides a feasible method to improve the processing speed of the CPU [7, 8]. The current widely used information processing method adopts photoelectric integration, that all-optical calculation part uses photons to carry information, which is transformed from electrical signals by photoelectric conversion. Then, after the calculation of the all-optical arithmetic part, the information is carried by electrical signals through photoelectric conversion [9]. Traditional all-optical calculation parts are designed primarily using regular and periodic structure, such as micro-ring resonant [10, 11], photonic crystal (PC) [12, 13], surface plasmon polaritons (SPPs) [14, 15], metamaterial [16, 17], etc. Zhu et al. [18] performed optical spatial differentiation computing at a single metal-dielectric interface and Nejad et al. [19] achieved analog computing with metamaterials. In addition, machine learning and deep learning based on non-Von Neumann architecture is also a popular method in optical parallel computing [20], [21], [22]. However, such structures usually need a large size, usually reach hundreds of microns, making it a bottleneck that can’t replace electrons. Although the size of SPPs circuits is small, their huge transmission loss is still a tremendous difficulty to limit the realization of low energy consumption. High-density integration requests strong interaction between light and matter, thus increasing the transmission and scattering loss. Inverse design method [23, 24] is an effective way to meet the demand of low energy consumption. Besides, traditional design methods are difficult to achieve high contrast “0” and “1” operations. Inverse design method provides a new design idea for the all-optical calculation with high transmittance and “0” “1” contrast. However, until now, it is still a huge challenge to realize ultrafast, ultra-low energy consumption and ultra-high-capacity data all-optical calculation, including addition, subtraction, multiplication and division at the same time in a photonic chip. Here, we propose a new encoding scheme for all-optical binary computation, including *n*-bit addition, subtraction, multiplication and division and experimentally demonstrate 1 bit calculation. The computation part includes a half binary adder and a shifter, whose feature sizes are only 2 μm × 19.5 μm and 4 μm × 9 μm, respectively. The half binary adder and shifter consist of three low-loss basic devices through inverse design method, which achieves the transmittance of each output waveguide greater than 90%. The distance between two adjacent basic devices is smaller than 1.5 μm, within wavelength magnitude scale. We have designed an all-optical calculator by inverse design method which greatly reduces the overall size of the device. The response time is the propagation time of the signal light in a single device, within 100 fs. The threshold energy consumption is within 10 fJ/bit, which is equal to the energy of signal light. Moreover, we theoretically present the *n*-bit calculation. Our results provide a new idea to realize ultrafast, ultra-low energy consumption and ultra-high-capacity data processing abilities all-optical *n*-bit binary computing.

## 2 Methods

### 2.1 Adjoint method

All-optical computing parts are mainly constructed by regular and periodic nanostructures. Traditionally, the designs of nanostructures are mainly based on finite difference time domain method (FDTD) and finite element method (FEM) by solving the maxwell’s equations, but it usually involves a long process through repeated simulation to get optimized results by manually adjusting the parameters of nanostructures, such as the width of the waveguides, the diameter of air holes and the size of the micro-rings [25]. Inverse design method, taking place repeating steps with algorithm technique to calculate unknown optical structures, is more suitable for the design and optimization of optical nanostructures [7, 26, 27], thus saving a lot of simulation time and break through the optimization effect of traditional methods. Adjoint method is most commonly used in the inverse design process, which is to solve the minimum value along the direction of gradient descent [28, 29]. It uses algorithms controlling the computer to find a dielectric structure that satisfies known specific characteristics such as desired electromagnetic field distribution or device function. In general, low loss and high transmittance are basic starting points for the design of our devices. We improve the adjoint method by equivalent the four waveguides in the form of “Pauli matrix” (see Section S1 in Supplementary material) and have designed three low-loss devices, including shifters, crossers and beam splitters. The whole designed platform was fabricated on Silicon-On-Insulator (SOI) with a 220-nm-thick silicon film on SiO_2_ substrate. The thickness of the device layer was 220 nm. The inverse design region was 2 μm × 2 μm × 220 nm, including 50 × 50 identical units, the size of each unit was 40 nm × 40 nm × 220 nm. Inverse design process used MATLAB to control simulation calculation through finite difference time domain software Lumerical FDTD Solutions. In the process of optimization, the refractive index of each unit continuously changed between the refractive index of silicon and air, and the final state of each unit become silicon or air. The working band was 1500–1600 nm. To simplify the calculation, we used the refractive index parameters at 1550 nm wavelength, the refractive indexes of silicon, air and substrate silica were set as $n_{Si}=3.46$ , $n_{air}=1$ and $n_{SiO_{2}}=1.47$ , respectively.

**Figure 1:**
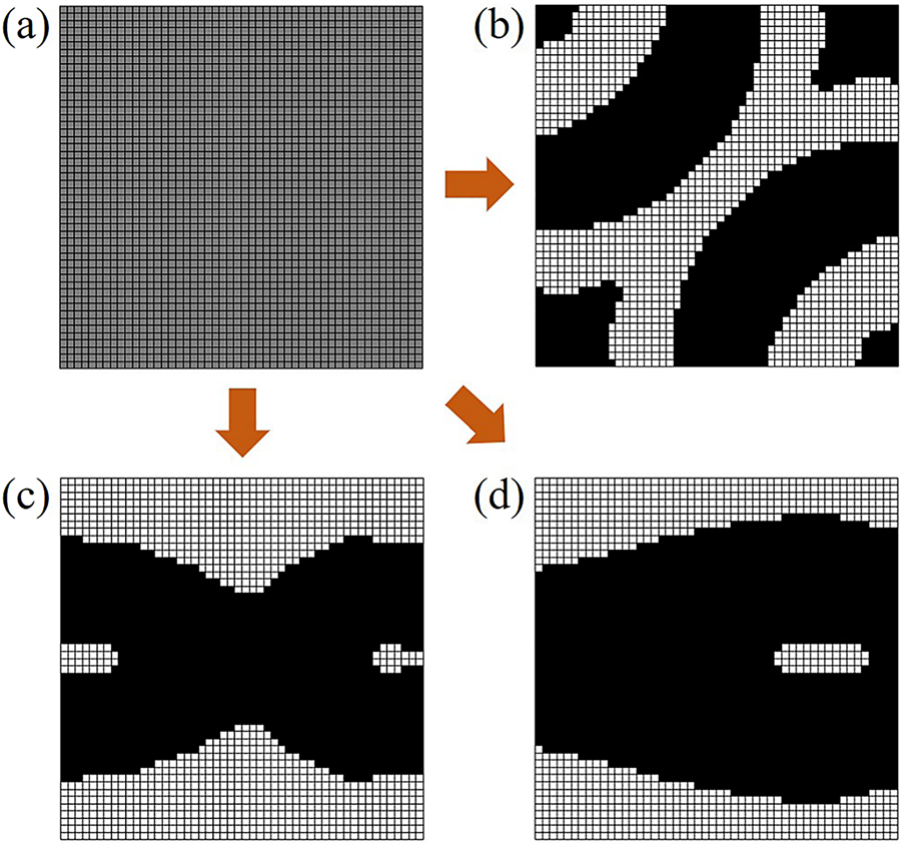
Design optimization process of the low-loss nanostructures. (a) The initialization permittivity distribution in the *x*–*y* two-dimensional cross-section, where bias = 0. (b) The discrete optimization permittivity distribution in the *x*–*y* two-dimensional cross-section of shifter structures and bias = infinity. (c) The discrete optimization permittivity distribution in the *x*–*y* two-dimensional cross-section of crossover structures and bias = infinity. (d) The discrete optimization permittivity distribution in the *x*–*y* two-dimensional cross-section of power splitter structures and bias = infinity.

The objective function was set as f=f(E(ϵ)) , whose independent variable was E(ϵ) . Specific derivation process was detailed in Section S2 in Supplementary material. The two-dimensional cross-section electric field distribution E was a function of permittivity distribution ϵ . The initial permittivity distribution was the intermediate value of the permittivity between silicon and air, as shown in Figure 1(a). Final optimized permittivity distribution asked for the objective function to meet the requirements of known specific characteristics. During the optimizing process, the permittivity of each unit changed continuously. Adjoint method required the permittivity to “drop one step” along the gradient descent direction, the gradient was calculated according to the objective function, and the permittivity was iterated along the gradient direction, given by Eq. (1).

(1) $$\epsilon_{i+1}=\epsilon_{i}-\alpha \frac{\partial f}{\partial \epsilon_{i}}$$

where, $\epsilon_{i}$ is the permittivity of the ith iteration, $\epsilon_{i+1}$ is the permittivity of the (i+1)th iteration, *α* is the descending step, $\alpha \frac{\partial f}{\partial \epsilon_{i}}$ varies between the refractive index values of Si and air, $\frac{\partial f}{\partial \epsilon_{i}}$ is the gradient to be calculated, and is expressed as Eq. (2).

(2) $$\frac{\partial f}{\partial \epsilon_{i}}=\frac{\partial E}{\partial \epsilon_{i}}\frac{\partial f}{\partial E}$$

where $\frac{\partial E}{\partial \epsilon_{i}}$ is the equivalent signal source and $\frac{\partial f}{\partial E}$ is the equivalent accompanying light source. We calculated the gradient value $G_{i}$ of the ith iteration, given by:

(3) $$G_{i}=\frac{\partial f}{\partial \epsilon_{i}}=-\omega^{2}\mu_{0}E^{\prime }E$$

where ω is the frequency of the signal light, $\mu_{0}$ is the vacuum permeability, $E^{\prime }$ is the two-dimensional cross-section electric field distribution of the accompanying light, E is the two-dimensional cross-section electric field distribution of the signal light. $G_{i}$ was get from $E^{\prime }$ and E to obtain the distribution of the dielectric constant of the (i+1)th iteration. When the predetermined number of iterations was reached, tended to the set square value of the refractive index of Si or air, which was 3.46 or 1. The bias factor β was set to change the refractive index of each basic unit along the final gradient direction, and the speed and magnitude of the change of the refractive index were controlled according to the bias factor. When β=infinity , we obtained the final discrete permittivity distribution of shifters, crossers and beam splitters, as shown in Figure 1(b)–(d), respectively.

### 2.2 The basic configuration

**Figure 2:**
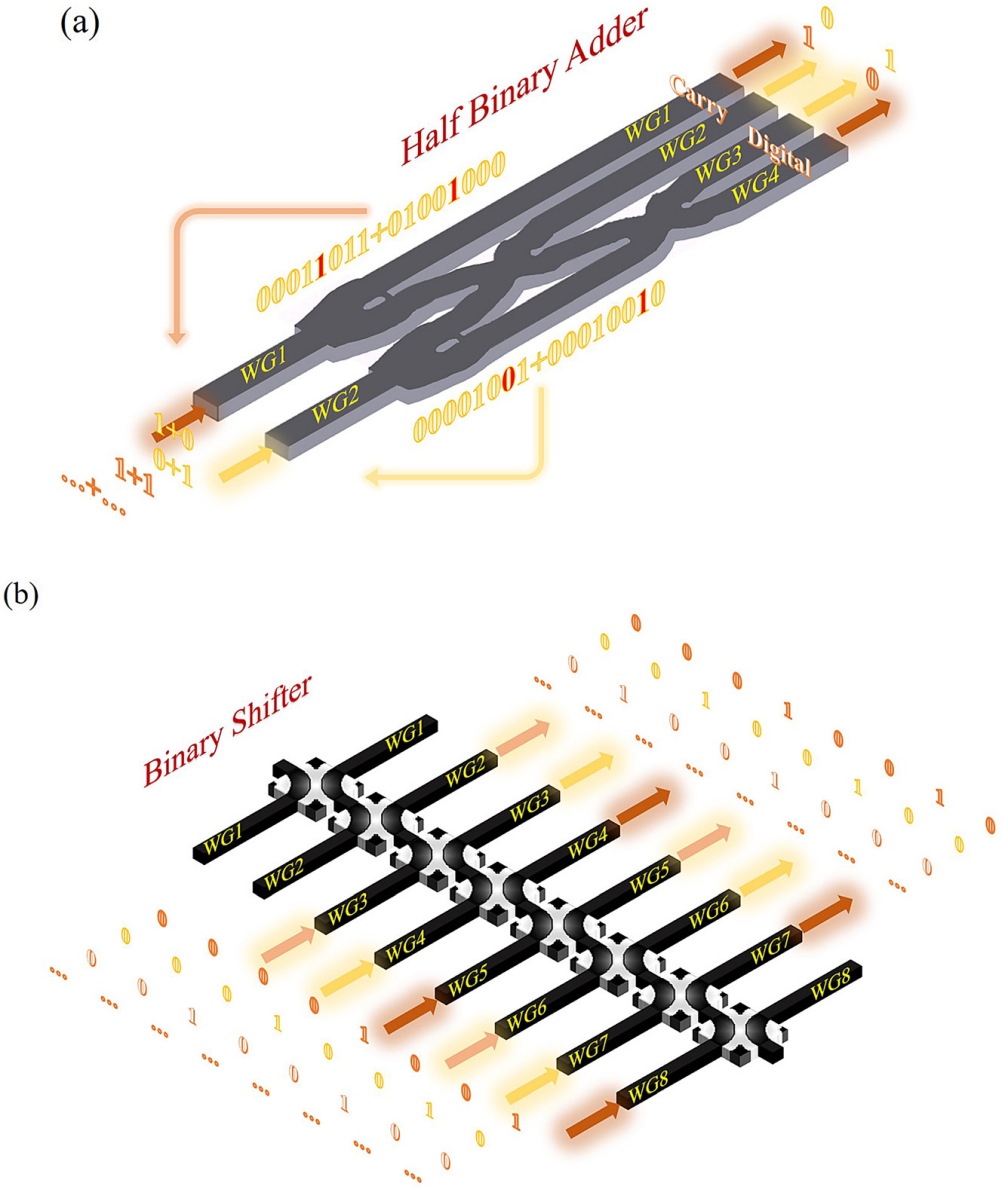
General configuration of the all-optical calculation part. (a) Configuration of the all-optical half binary adder. (b) Configuration of the all-optical binary shifter.

The all-optical computation part includes a half binary adder and a binary shifter. We combine two crossers with two beam splitters to form the half binary adder, whose basic configuration is shown in Figure 2(a). It includes two input waveguides and four output waveguides. Signal light is input in the form of coded pulsed light. The upper two waveguides represent the “Carry” bit and the lower two waveguides represent the “Digital” bit. The binary shifter is formed with eight shifters combined with eight input waveguides and eight output waveguides, as shown in Figure 2(b). The half-adder and shifter are on the same photonic chip, and the all-optical computation part is formed by the synergistic action of computer programming. The feature sizes of the half binary adder and the shifter are only 2 μm × 19.5 μm and 4 μm × 9 μm, respectively. The distance between two adjacent inverse-designed units is smaller than 1.5 μm, within wavelength magnitude scale.

### 2.3 Encoding scheme

#### 2.3.1 Half binary adder

A half adder is referred to an adder circuit that adds two input data bits and outputs a result bit and a carry bit. It is a realization of two binary number addition operation circuit. The half binary adder is formed with four inverse-design devices, as shown in Figure 2(a). We define the light of “1 + 0” or “0 + 1” to input in the upper waveguide, and the light of “1 + 1” to input in the lower waveguide. The input light comes from the light pulse signal programmed by the computer, which is generated by the electrical pulse signal through the photoelectric conversion process. The coded input signal light is determined by the feedback of the upper state and the input signal of the current state. We know that “1 + 0 = 01”, “0 + 1 = 01”, and “1 + 1 = 10”. We define the two output waveguides as one bit of a binary number, where waveguide 1 has light, waveguide 2 has no light is defined as “1”, and waveguide 1 has no light, and waveguide 2 has light is defined as “0”. Waveguide 3 has light, waveguide 4 has no light is defined as “1”, and waveguide 3 has no light, and waveguide 4 has light is defined as “0”. The half adder designed in this way can achieve arbitrary bits of binary addition. The encoding scheme of all-optical half binary adder is shown in Table 1.

**Table 1:** Encoding scheme of all-optical half binary adder.

Input states		Output states				Defined states	
WG1	WG2	WG1	WG2	WG3	WG4	Carry	Digital
1	0	1	0	0	1	1	0
0	1	0	1	1	0	0	1

#### 2.3.2 Binary shifter

The binary shifter is formed with eight inverse-design devices, as shown in Figure 2(b). The basic multi-bit binary multiplication is carried out through shifting bits. The input light comes from the light pulse signal programmed by the computer, which is generated by the electrical pulse signal through the photoelectric conversion process. We know that the result of shifting “00001001” 1 bit is “00010010”, the result of shifting “00001001” 2 bit is “00100100” and the result of shifting “00001001” 3 bit is “01001000”. We convert one digit of the multiplier into binary form and identify it from low bit to high bit. If it is “1”, the other digit of the binary multiplier moves forward by corresponding digits, and the gaps are filled with zero. If it is “0”, the multiplier does no processing. The shifter designed in this way can shift any number of bits according to the number of inverse-design devices. The configuration in Figure 2(b) can realize up to 8 bit shift operations of 8 bit binary numbers.

#### 2.3.3 Calculation ability

The maximum result that can be obtained from the structure is $2^{n-1}$ , the size of which is determined by the number of shifters n . In this paper, the number of shifters is 8, so we can get the maximum multiplication result is $2^{8-1}=127$ , which grows exponentially with n . Therefore, by increasing the number of shifters, we can theoretically compute arbitrary multiplication. If we add 16 devices, then the maximum result is 32,768. If 32 devices, then we can get the maximum result is $2\times 10^{9}$ . Our all-optical computing method has a huge advantage compared to the number of transistors in the billions.

### 2.4 Realization of all-optical calculation

**Figure 3:**
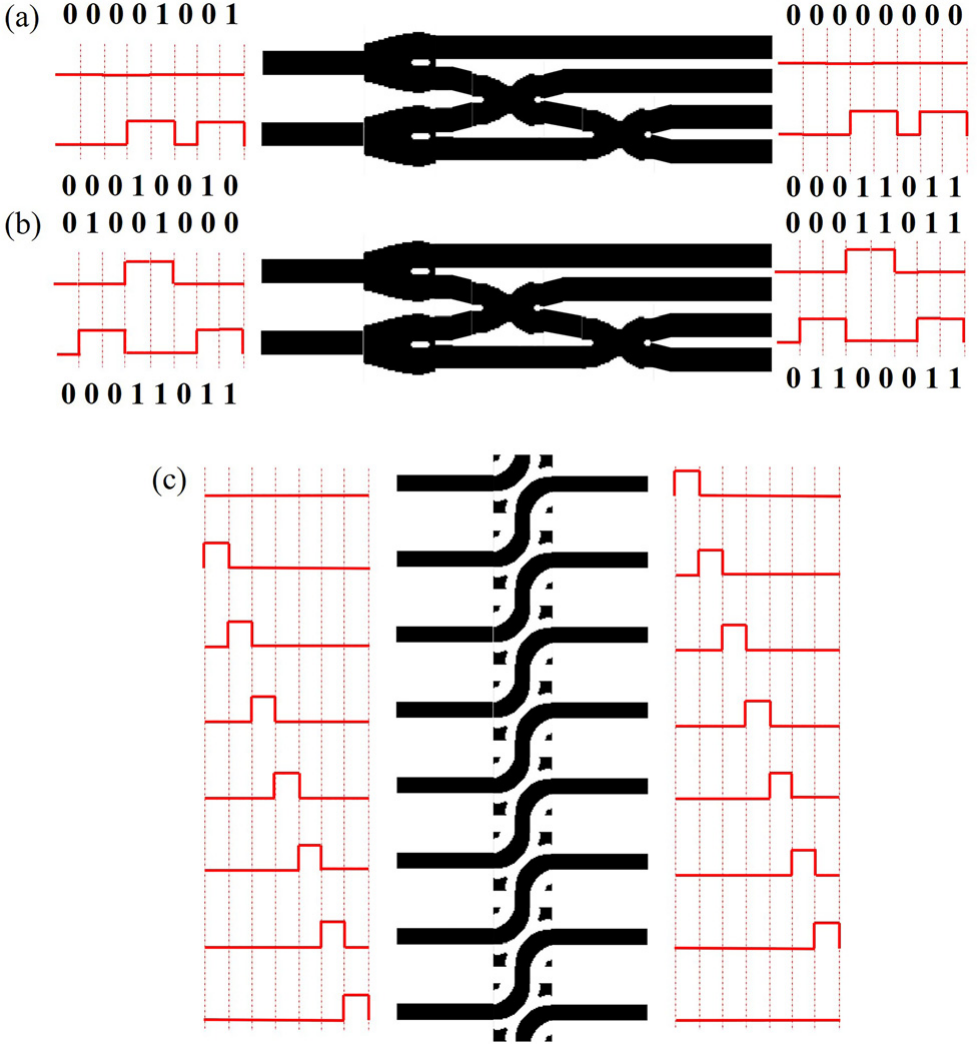
The basic working principle of the all-optical calculation part. (a) The “00001001 + 00010010” input state of signal light in the form of light pulse. (b) The “01001000 + 00011011” input state of signal light in the form of light pulse. (c) The results of shifter when signal lights are input into each waveguide in turn.

To clearly show the operating principle of our all-optical computation part, we used MATLAB coding to design the signal light input (see Section S3 in Supplementary material). We take the 9 × 11 calculation as an example. First, we convert 9 and 11 into 8 bit binary numbers, which are “00001001” and “00001011”, respectively. We need three-time shifting and the result of shifting 1 bit is “00010010”, 2 bit is “00100100” and 3 bit is “01001000”. Then we calculate the addition part, signal light “00001001” and the result of shifting 1 bit “00010010” is input in the form of pulsed light. After transmitting in the half binary adder, the carry bits are “00000000” and the digital bits are “00011011”, then we get the right result of “00001001 + 00010010 = 00011011”, as shown in Figure 3(a). Next, we calculate the second addition part, signal light “00011011” and the result of shifting 3 bit “00010010” is input in the form of pulsed light. After transmitting in the half binary adder, the carry bits are “00011011” and the digital bits are “01100011”, then we get the right result of “01001000 + 00011011 = 01100011”, as shown in Figure 3(b). The shifting operation can also be achieved in the form of coded pulsed light, as shown in Figure 3(c). Finally, we convert “01100011” into decimal numbers, which is “99”, the right result of “9 × 11”.

## 3 Results and discussion

### 3.1 Simulation results

**Figure 4:**
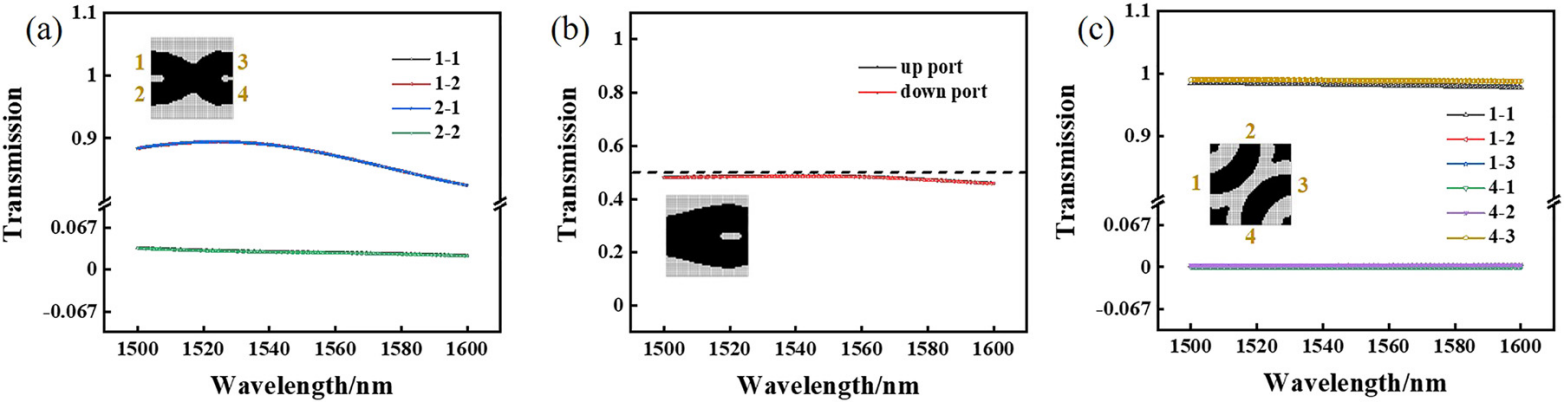
Simulation results of the normalized transmission of low-loss devices. (a) Transmission of crosser in the range of wavelength 1500–1600 nm. (b) Transmission of power splitter in the range of wavelength 1500–1600 nm. (c) Transmission of shifter in the range of wavelength 1500–1600 nm.

Low loss and high transmittance devices have always been our goals. The transmittance of crossers, shift devices and beam splitter devices by inverse design method is around 90%. As shown in Figure 4(a), when the signal light is input in WG1 of the crosser, the transmission in WG4 is 90% at 1550 nm and greater than 80% at 1500–1600 nm in a wide band range, when the signal light is input in WG2 of the crosser, the transmission in WG3 is 90% at 1550 nm and greater than 80% at 1500–1600 nm in a wide band range. As shown in Figure 4(b), the transmittance of the upper and lower waveguides of the beam splitter is 49% at 1550 nm, the sum of them is 98%, and the transmittance in a wide band range of 1500–1600 nm is more than 90%. As shown in Figure 4(c), when the signal light is input in WG1 of the crosser, the transmission in WG2 is 98% at 1550 nm, and the transmittance is greater than 98% in a wide band range of 1500–1600 nm. When the signal light is input in WG4 of the crosser, the transmission in WG3 is 98% at 1550 nm, and the transmittance is greater than 98% in a wide band range of 1500–1600 nm.

**Figure 5:**
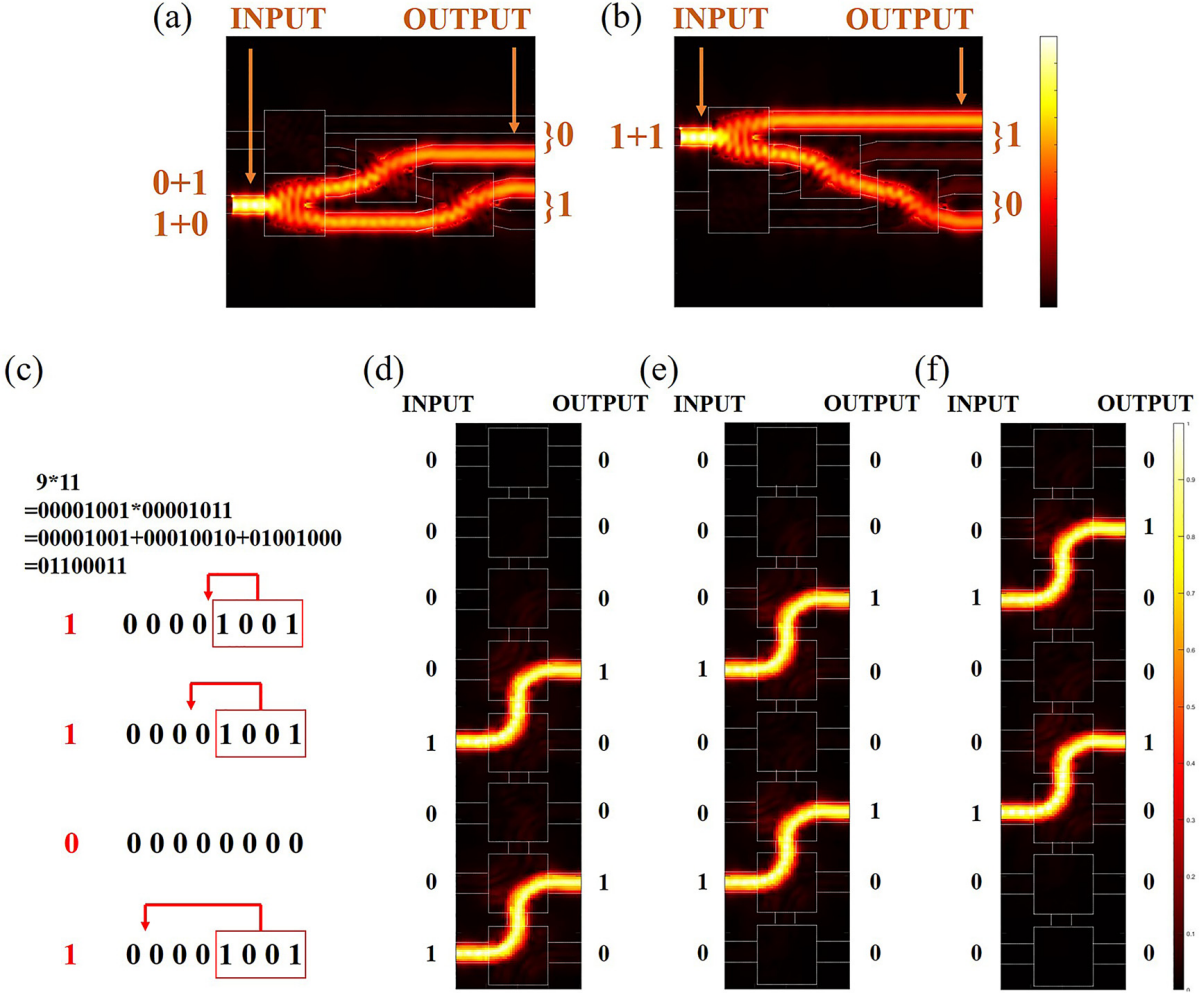
Simulation results of all-optical calculation part. (a) The “0 + 1” or “1 + 0” input states of normalized intensity distribution in the *x*–*y* plane from theoretical calculation. (b) The “1 + 1” input state of normalized intensity distribution in the *x*–*y* plane from theoretical calculation. (c) The basic working principle of the all-optical binary shifter. (d) The “00001001” states of normalized intensity distribution in the *x*–*y* plane from theoretical calculation, the output result is “00010010”. (e) The “00010010” states of normalized intensity distribution in the *x*–*y* plane from theoretical calculation, the output result is “00100100”. (f) The “00100100” states of normalized intensity distribution in the *x*–*y* plane from theoretical calculation, the output result is “01001000”.

**Figure 6:**
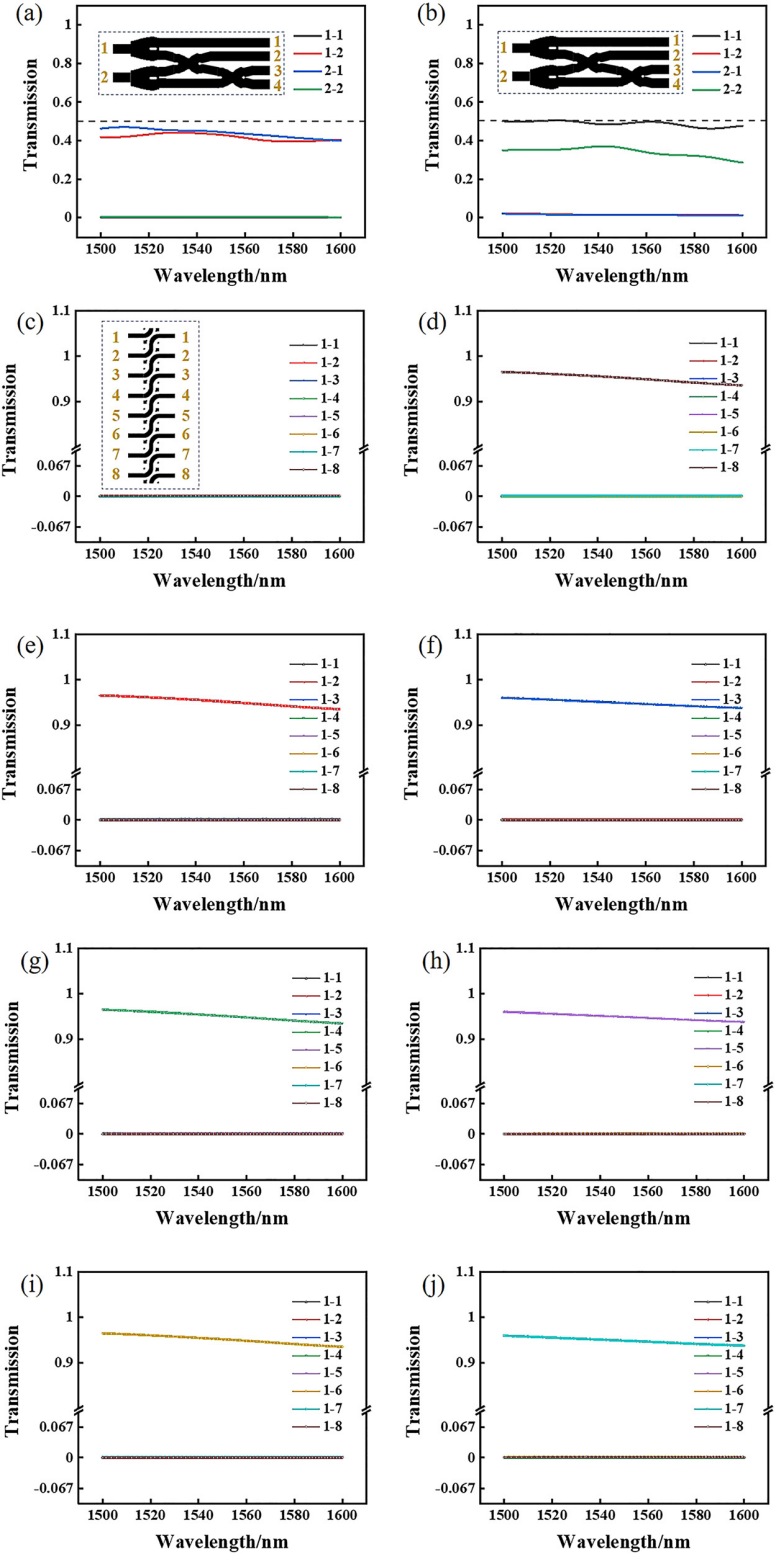
Simulation results of the normalized transmission of all-optical calculation part. (a) Transmission of four output waveguides of “0 + 1” or “1 + 0” input states in the range of wavelength 1500–1600 nm. (b) Transmission of four output waveguides of “1 + 1” input state in the range of wavelength 1500–1600 nm. (c)–(j) are the transmission of eight output waveguides of “10000000”, “01000000”, “00100000”, “00010000”, “00001000”, “00000100”, “00000010” and “00000001” input states in the range of wavelength 1500–1600 nm, respectively.

Next, based on the above coding mechanism, we calculate the simulation results of the half binary adder. As shown in Figure 5(a), when the signal light is input in the lower waveguide, it outputs in WG2 and WG3, corresponding to “0 + 1” or “1 + 0” input. As shown in Figure 5(b), when the signal light is input in the upper waveguide, it outputs in WG1 and WG4, corresponding to “1 + 1” input. As shown in Figure 6(a), when the signal light is input in the lower waveguide, the transmittance of WG1 is 0.1% at 1550 nm, as shown in the black line in Figure 6(a), the transmittance of WG2 is 45% at 1550 nm, as shown in the red line in the figure, and the contrast is 26.5 dB. The transmittance of WG3 is 43% at 1550 nm, as shown in the blue line in the figure; the transmittance of WG4 is 0.1% at 1550 nm, as shown in the green line in the figure, and the contrast is 26.3 dB. The total transmittance of the 2 and 3 waveguides is 88%, which meets the high transmittance requirement of our demand. As shown in Figure 6(b), when the signal light is input in the upper waveguide, the transmittance of WG1 is 49% at 1550 nm, as shown in the black line in Figure 6(a), the transmittance of WG2 is 0.1% at 1550 nm, as shown in the red line in the figure, and the contrast is 26.9 dB. The transmittance of WG3 is 0.1% at 1550 nm, as shown in the blue line in the figure; the transmittance of WG4 is 38% at 1550 nm, as shown in the green line in the figure, and the contrast is 25.8 dB. The total transmittance of the WG2 and WG3 is 87%, which meets the high transmittance requirement of our demand.

Similarly, we calculate the simulation results of the shifter. The signal light is input in the left waveguide shift 1 bit and output in the right waveguide. As shown in Figure 5(d)–(f), when the signal light input in WG5 and WG8, WG4 and WG7, WG3 and WG6, it output in WG4 and WG7, WG3 and WG6, WG2 and WG5, respectively. When the signal light input in WG1, WG2, WG3, WG4, WG5, WG6 and WG7 on the left waveguides, the signal light mainly output in WG2, WG3, WG4, WG5, WG6, WG7 and WG8 on the right waveguides, respectively. The transmittance of the eight output waveguides on the right under different input waveguides is shown in Figure 6. In Figure 6(c), when signal light is input in WG1 on the left, the transmittance of all waveguides is less than 0.1%. In Figure 6(d)–(j), when signal light is input in WG2, WG3, WG4, WG5, WG6, WG7 and WG8 on the left, the corresponding high transmittance of WG1, WG2, WG3, WG4, WG5, WG6 and WG7 on the right is more than 95%, the transmittance of other waveguides is less than 0.1%, and the contrasts of high transmittance and low transmittance are more than 29.8 dB.

Finally, we calculated the response time and the threshold energy consumption of our all-optical calculation part. The footprint of a single device was 2 μm × 2 μm, so for the calculation part, the propagation time of the signal light was the response time. The theoretical response time was 23 fs, within 100 fs. (see Section S3 in Supplementary material). The threshold energy consumption is within 10 fJ/bit (see Section S4 in Supplementary material).

### 3.2 Experiment result

**Figure 7:**
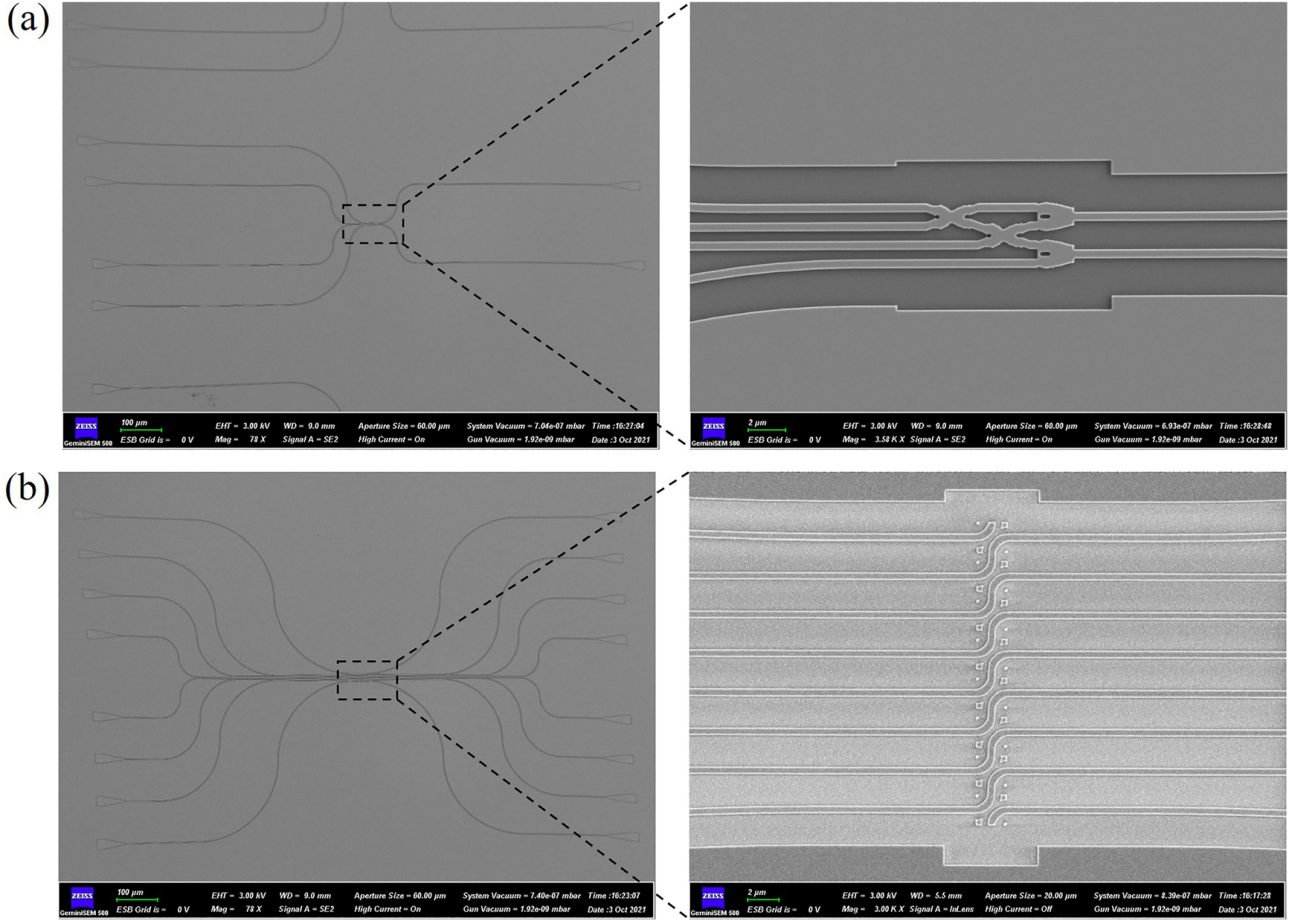
SEM image of the all-optical calculation part. (a) SEM image of the all-optical half binary adder. (b) SEM image of the all-optical binary shifter.

**Figure 8:**
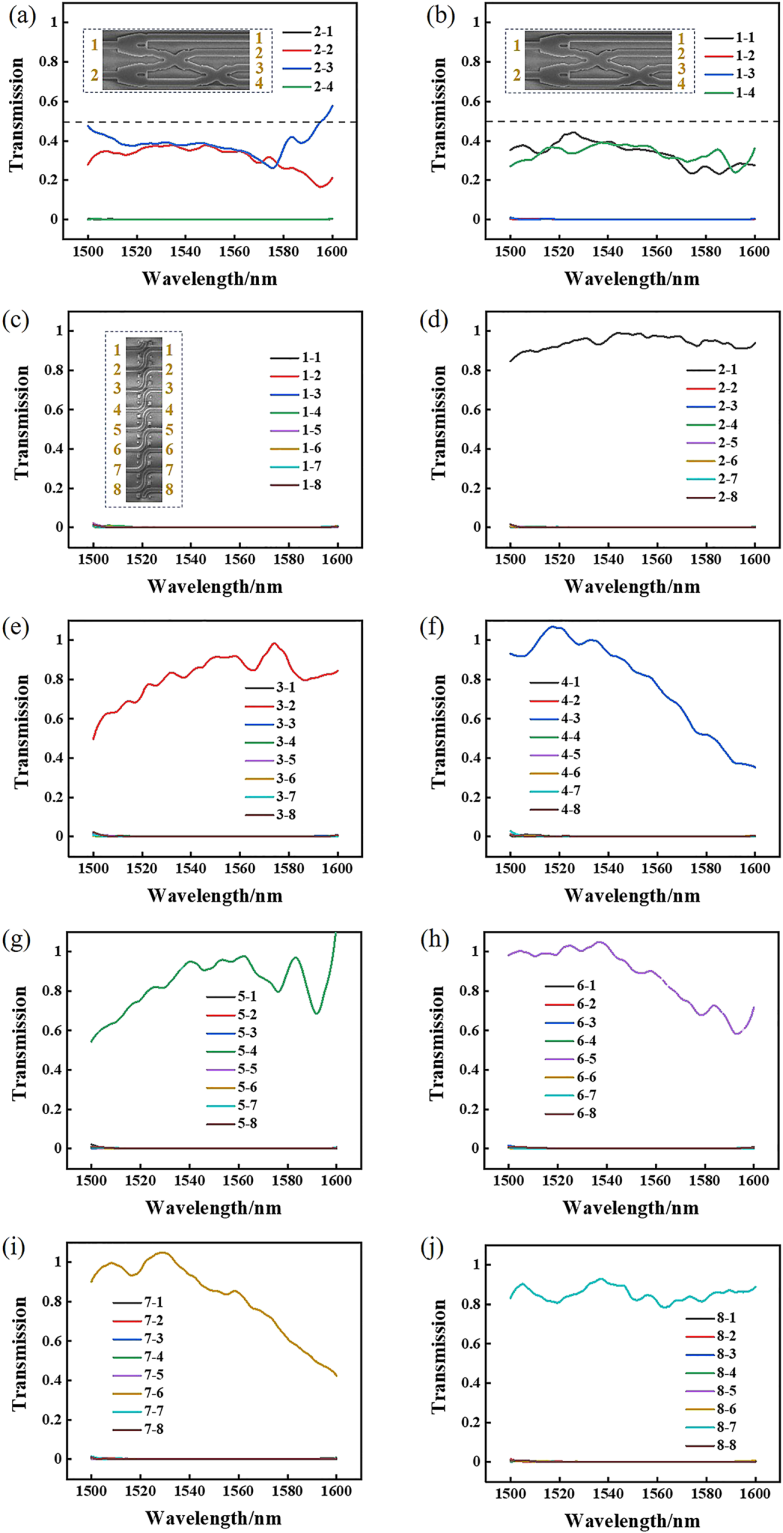
Experiment results of the normalized transmission of all-optical calculation part. (a) Transmission of four output waveguides of “0 + 1” or “1 + 0” input states in the range of wavelength 1500–1600 nm. (b) Transmission of four output waveguides of “1 + 1” input state in the range of wavelength 1500–1600 nm. (c)–(j) are the transmission of eight output WGs of “10000000”, “01000000”, “00100000”, “00010000”, “00001000”, “00000100”, “00000010” and “00000001” input states in the range of wavelength 1500–1600 nm, respectively.

The all-optical calculation part was fabricated using electron beam lithography system. The scanning electron microscopy (SEM) image of the completed device is analyzed through Zeiss GeminiSEM 500, as shown in Figure 7. Figure 7(a) shows the SEM of all-optical half binary adder. Figure 7(b) shows the SEM of all-optical binary shifter. We measure the linear transmission spectrum of the devices using an optical fiber coupling system. We have experimentally tested the results of the half binary adder. As shown in Figure 8(a), when the signal light is input in the lower waveguide, the transmittance of WG1 is 39%, as shown in the black line, the transmittance of WG2 is 0.1%, as shown in the red line, and the contrast ratio is 25.9 dB. The transmittance of WG3 is 0.1%, as shown in the blue line in the figure, the transmittance of WG4 is 36%, as shown in the green line in the figure, the contrast ratio of high transmittance and low transmittance is 25.6 dB. The total transmittance of WG1 and WG4 is 75%, which meets the high transmittance requirement of our device itself. As shown in Figure 8(b), when the signal light is input in the lower waveguide, the transmittance of WG1 is 36%, as shown in the black line, and the transmittance of WG2 is 0.1%, as shown in the red line, and the contrast ratio is 25.6 dB. The transmittance of WG3 is 0.1%, as shown in the blue line in the figure, the transmittance of WG4 is 0.1%, as shown in the green line in the figure; the contrast ratio is 25.9 dB. The total transmittance of the WG1 and WG4 is 88%, which also meets the high transmittance requirement of our device itself.

Similarly, we measure the transmittance results of the shifter. The signal light is input in the left waveguide shift 1 bit and output in the right waveguide. When the signal light is input in WG1, WG2, WG3, WG4, WG5, WG6 and WG7 on the left waveguides, the signal light is mainly output in WG2, WG3, WG4, WG5, WG6, WG7 and WG8 on the right waveguides. The transmittance of the eight output waveguides on the right under different input waveguides is shown in Figure 8. In Figure 8(c), when signal light is input in WG1 on the left, the transmittance of all waveguides is less than 0.1%. In Figure 8(d)–(j), when signal light is input in WG2, WG3, WG4, WG5, WG6, WG7 and WG8 on the left, the corresponding high transmittance of WG1, WG2, WG3, WG4, WG5, WG6 and WG7 on the right is 95%, 85%, 80%, 90%, 90%, 82% and 80%, respectively. The transmittance of other waveguides is all less than 0.1%, and the contrast of high transmittance and low transmittance is more than 29.0 dB. The experimental results are in agreement with the simulation results.

## 4 Conclusions

In conclusion, we propose a new encoding scheme for all-optical binary computation, including *n*-bit addition, subtraction, multiplication and division. We theoretically present the *n*-bit calculation and experimentally demonstrate 1 bit calculation. The computation part includes a half binary adder and a shifter which achieve small feature size of only 2 μm × 19.5 μm and 4 μm × 9 μm, respectively. We have designed an all-optical calculator by inverse design method which greatly reduces the overall size of the device. The half binary adder and shifter consist of three low-loss basic devices through inverse design method, which achieves the transmittance of each output waveguide greater than 90%. The distance between two adjacent basic devices is smaller than 1.5 μm, within wavelength magnitude scale. The response time is the propagation time of the signal light in a single device, within 100 fs. The threshold energy consumption is within 10 fJ/bit, which is equal to the energy of signal light. Our results provide a new idea to realize ultrafast, ultra-low energy consumption and ultra-high-capacity data processing abilities all-optical *n*-bit binary computing.

## Supplementary Material

Supplementary Material

Supplementary Material
