# Supplementary material for: All-optical binary computation based on inverse design method
Source: Nanophotonics. 2021 Oct 26;11(9):2117–27. doi: 10.1515/nanoph-2021-0467 (PMC11501324; doi:10.1515/nanoph-2021-0467)
Supplement: Supplementary file 1 — Supplementary Material [file j_nanoph-2021-0467_suppl_001.docx]

**SUPPORTING INFORMATION**

All-Optical Binary Computation Based on Inverse Design Method

*Huixin Qi, Zhuochen Du, Jiayu Yang, Xiaoyong Hu*, Qihuang Gong*

**Section S1. Design principles of low loss devices**

**Matrix representation of optical devices**

An optical device has $n$input ports and $m$ output ports (some ports may be both input and output). Devices made of linear materials, the electric field at the output ports and the input ports of which are related by Matrix:

| $\left[ \begin{aligned} b_{1} \\ \vdots\\ b_{m} \end{aligned} \right]=\left[ \begin{matrix} F_{11} & \cdots& F_{1n} \\ \vdots& \ddots& \vdots\\ F_{n1} & \cdots& F_{nn} \end{matrix} \right]\left[ \begin{aligned} a_{1} \\ \vdots\\ a_{n} \end{aligned} \right]$ | (S1) |
| --- | --- |

where, $a_{i}$ represents the electric field at $i^{th}$ input waveguides, $b_{i}$ represents the electric field at $i^{th}$ output waveguides. $a_{i},b_{i},F_{ij}$ are all complex numbers, which can be abbreviated as

| $\left\vert\left. b \right\rangle=\hat{F} \right\vert\left. a \right\rangle$ | (S2) |
| --- | --- |

Here we consider only the waveguide fundamental mode (or near) transmitted in the input or output waveguide. The total input power is normalized:

| $\left\langle a \vert a \right\rangle=1$ | (S3) |
| --- | --- |

**The limitation of energy conservation**

Due to the limitation of energy conservation, not any matrix $F$ can be realized. The output power cannot become larger than 1:

| $\left\langle b \vert b \right\rangle\leq1$  $\left\langle a\vert\hat{F}^{\dagger}\hat{F}\vert a \right\rangle\leq1$ | (S4) |
| --- | --- |

We define $\hat{G}=\hat{F}^{\dagger}\hat{F}$. It is easy to prove that $\hat{G}$ is Hermitian ($\hat{G}^{\dagger}=\left( \hat{F}^{\dagger}\hat{F} \right)^{\dagger}=\hat{F}^{\dagger}\left( \hat{F}^{\dagger} \right)^{\dagger}=\hat{F}^{\dagger}\hat{F}=\hat{G}$). All $n$ eigenvalues of $\hat{G}$ are real and positive semidefinite. Conservation of energy is satisfied if the maximum eigenvalue of $\hat{G}$ is less than or equal to 1. We can also represent it in terms of the norm of the following matrix:

| $\left\Vert F \right\Vert_{2}\leq1$ | (S5) |
| --- | --- |

$\hat{F}$ of Eq. (S5) is achievable.

**Section S2. Adjoint method**

We start with Maxwell's equations

| $\nabla\times\boldsymbol{E}=-\frac{\partial\boldsymbol{B}}{\partial t}$ | (S6) |
| --- | --- |
| $\nabla\cdot\boldsymbol{D}=\rho$ | (S7) |
| $\nabla\cdot\boldsymbol{B}=0$ | (S8) |
| $\nabla\times\boldsymbol{H}=\boldsymbol{J}+\frac{\partial\boldsymbol{D}}{\partial t}$ | (S9) |

Considering the region of anisotropic, non-ferromagnetic medium without free charge, the equations are written in the frequency domain

| $\nabla\times\boldsymbol{E}=i\omega\boldsymbol{B}$ | (S10) |
| --- | --- |
| $\nabla\cdot\left( \varepsilon\boldsymbol{E} \right)=0$ | (S11) |
| $\nabla\cdot\boldsymbol{B}=0$ | (S12) |
| $\nabla\times\boldsymbol{B=}\mu_{0}\boldsymbol{J-}i\omega\varepsilon\mu_{0}\boldsymbol{E}$ | (S13) |

Substitute Eq.(S10) into Eq. (S13), we can get Eq.(S14) and Eq.(S15)

| $\nabla\times\left( -\frac{i}{\omega}\nabla\times\boldsymbol{E} \right)=\mu_{0}\boldsymbol{J}-i\omega\varepsilon\mu_{0}\boldsymbol{E}$ | (S14) |
| --- | --- |
| $\nabla\times\left( \nabla\times\boldsymbol{E} \right)-\omega^{2}\varepsilon\mu_{0}\boldsymbol{E}=i\omega\mu_{0}\boldsymbol{J}$ | (S15) |

In numerical calculation, space is discretized. Assume that the region is discretized into n small regions, then Eq.(S15) can be written as

| $AE\boldsymbol{=}i\omega\mu_{0}J$ | (S16) |
| --- | --- |

where $E\boldsymbol{\in}C^{n\times1}$**,** $J\boldsymbol{\in}C^{n\times1}$, A represents the operator $\nabla\times\left( \nabla\times\right)-\omega^{2}\varepsilon\mu_{0}$ in the discrete space matrix representation *(*$R^{n\times n}$*)*, the $i^{th}$ element represents A certain rectangular coordinate component of the electric field value of the $i^{th}$ element，the $i^{th}$ element represents A certain rectangular coordinate component of the conduction current density of the $i^{th}$ element, which can be set as source of excitation electromagnetic field.

Our target function can always be defined in terms of the (electric) field. An intuitive example is that we want to maximize the density of electromagnetic field energy flow through a certain section, then the objective function can be written as

| $f=\int_{s} \left( \boldsymbol{E}\times\boldsymbol{H} \right)\boldsymbol{\cdot}d\boldsymbol{S}=\sum\left( \boldsymbol{E}\times\boldsymbol{H} \right)\boldsymbol{\cdot}\Delta\boldsymbol{S}$ | (S17) |
| --- | --- |

In electromagnetic waves, $H\propto E$ (only the norm is proportional), then the objective function can be written (ignoring the proportionality coefficient).

| $f=\sum\boldsymbol{E}^{\boldsymbol{2}}\Delta S$ | (S18) |
| --- | --- |

For such problems, the electric field $\boldsymbol{E}$ is the independent variable of the objective function, i.e

| $f=f\left( \boldsymbol{E} \right)$ | (S19) |
| --- | --- |

In the optimization problem, our goal is to find the distribution of the dielectric constant of the region to minimize the objective function.

To keep the problem simple, we first set the permittivity of each unit to be continuously varying. According to the idea of gradient descent algorithm, we only need to let the dielectric constant "drop one step" along the gradient direction to make the objective function smaller

| $\varepsilon=\varepsilon-\alpha\frac{\partial f}{\partial\varepsilon}$ | (S20) |
| --- | --- |

Here $\varepsilon\boldsymbol{\in}R^{n\times1}$**,** the $i^{th}$element represents the dielectric constant of the unit, $\alpha$ is the step length, $\frac{\partial f}{\partial\varepsilon}\boldsymbol{\in}R^{n\times1}$, the $i^{th}$element represents $\frac{\partial f}{\partial\varepsilon_{i}}$。Notice that the objective function does not explicitly contain $\varepsilon$. Computed $\frac{\partial f}{\partial\varepsilon}$ , we need

| $\frac{\partial f}{\partial\varepsilon}=\frac{\partial E}{\partial\varepsilon}\frac{\partial f}{\partial E}$ | (S21) |
| --- | --- |

$\frac{\partial E}{\partial\varepsilon}\boldsymbol{\in}C^{n\times n}$, the $i^{th}$ row and $j^{th}$column represents $\frac{\partial E_{j}}{\partial\varepsilon_{i}}$, $\frac{\partial f}{\partial E}\boldsymbol{\in}C^{n\times1}$, the $i^{th}$element represents $\frac{\partial f}{\partial E_{i}}$. Because the dependence of $f$ on the electric field is defined by us, so $\frac{\partial f}{\partial E}$ can be figured out by definition. Note that $f$ can depend on the three right-angled components of the electric field. The $\boldsymbol{E}$ in Eq.(S21) represents only one right-angled component, and the rest of the terms are of the same form. The key point of the problem lies in the calculation of $\frac{\partial E}{\partial\varepsilon}$. To make the form clear, the matrix and vector are written by the way of index and the Einstein summation convention is adopted.

| $A_{kj}E_{j}\boldsymbol{=}i\omega\mu_{0}J_{k}$ | (S22) |
| --- | --- |

Take the partial derivative of both sides for$\varepsilon_{i}$

| $\frac{\partial A_{kj}}{\partial\varepsilon_{i}}E_{j}+A_{kj}\frac{\partial E_{j}}{\partial\varepsilon_{i}}=0$  $\frac{\partial E_{j}}{\partial\varepsilon_{i}}=\left( A^{-1} \right)_{kj}\frac{\partial A_{kl}}{\partial\varepsilon_{i}}E_{l}$  $\frac{\partial A_{kl}}{\partial\varepsilon_{i}}=-\omega^{2}\mu_{0}\delta_{ik}\delta_{kl}$  $\frac{\partial E_{j}}{\partial\varepsilon_{i}}=-\omega^{2}\mu_{0}\left( A^{-1} \right)_{ij}E_{i}$  $\frac{\partial f}{\partial\varepsilon_{i}}=\frac{\partial E_{j}}{\partial\varepsilon_{i}} \frac{\partial f}{\partial E_{j}}=-\omega^{2}\mu_{0}\left( A^{-1} \right)_{ij}E_{i}\frac{\partial f}{\partial E_{j}}$  $\frac{\partial f}{\partial\varepsilon_{i}}=-\omega^{2}\mu_{0}\left( A^{-1}\frac{\partial f}{\partial E} \right)_{i}E_{i}$ | (S23) |
| --- | --- |

Considering that we have figured $\frac{\partial f}{\partial E}$, we can take it as an equivalent source, i.e

| $i\omega\mu_{0}J^{'}=\frac{\partial f}{\partial E}$ | (S24) |
| --- | --- |

It is obtained by numerical calculation $E^{'}=A^{-1}(i\omega\mu_{0}J^{'})$. The gradient can be expressed as

| $\frac{\partial f}{\partial\varepsilon_{i}}=-\omega^{2}\mu_{0}E_{i}^{'}E_{i}$ | (S25) |
| --- | --- |

Therefore, we only need to perform two numerical calculations to get the field distribution, and then we can get the gradient of the objective function for the dielectric constant.

**Section S3. Input signal encoding control method**

**MATLAB control program**

In order to clearly show the operating principle of our all-optical computing unit, we used MATLAB coding to design the signal light input. Signal light is input in the form of pulsed light. Figure S1(a) shows the input interface, operation instruction interface, the calculation steps interface and result interface of MATLAB control program. We take the 9×11 calculation as an example. The operation instruction interface is “times” and the calculation steps are shown in the calculation steps interface. The calculation steps are shown as follows: first we convert 9 and 11 into 8-bit binary numbers, which are “00001001” and “00001011”, respectively. We need three-time shifting and the result of shifting 1-bit is “00010010”, 2-bit is “00100100” and 3-bit is “01001000”, as shown in Figures (b)(c)(d), respectively. Then we calculate the addition part, “01001000+00010010=01011010” and “01011010+00001001=01100011”, which is operated in Figures (e)(f). Finally, we convert “01100011” into decimal numbers, which is “99”, the right result of “9×11”.

**Experiment control method**

In order to input the encoded signal light into our designed structure, the electro-optic modulator was needed in the experiment. We replaced this process of electrical modulation coding with a program in theoretical calculation. We presented multi-bits calculation theoretically and only demonstrated 1-bit of calculation in the experiment. In actual measurements, electro-optic modulation was a slower process than optical computation, usually reaching hundred Mbit/s, while the speed of the optical computation part was 1Tbit/s. The current on-chip CMOS-compatible electro-optical modulators have modulation speed of up to 10 Gbit/s, which also have a big difference from the all-optical computing part. At present, all-optical modulators can achieve modulation speeds of 1 Tbit/s. In the next work, we added the all-optical modulation part before the optical calculation part. The response time of all-optical modulation is 1 Tbit/s, which can solve the response time difference of 10^4^ magnitude between electro-optical modulation and all-optical calculation part.


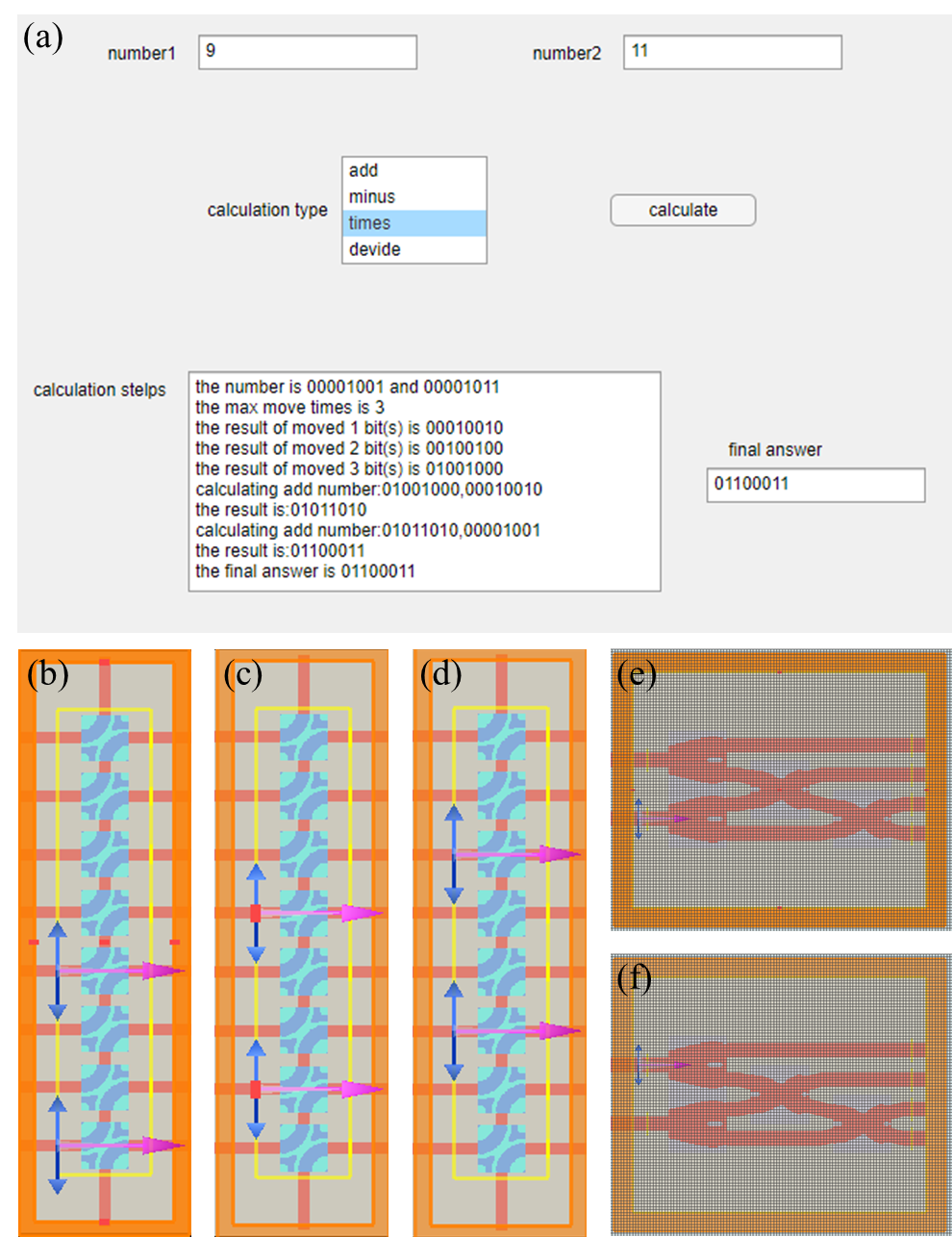


Figure S1: Process of MATLAB control program. (a) Input interface, operation instruction interface, the calculation steps interface and result interface of MATLAB control program. (b)(c) and (d) are software interface in calculating the shifting part of “00001001”, “00010010” and “00100100”, respectively. (e) and (f) are software interface in calculating the addition part of “0+1, 1+0” and “1+1”, respectively.

**Section S4. Experiment**

**Fabrication**

We first clean SOI piece (2 cm×2 cm) at 70°C both DSF-2 and DQY-2 bath for 60 min. The integrated photonic devices and waveguides were defined by patterning a 250 nm ZEP-520A resist layer using JEOL JBX-9500FS electron beam lithography system. The etching process of silicon device layer uses equipment OXFORD PlasmaPro 100 Cobra 180. Then we strip resist in 70°C DQY-2 baths for 30 min. At this point, the whole device preparation process is over.

**Measurement**

The scanning electron microscopy (SEM) image of the completed device is analyzed through Zeiss GeminiSEM 500. We measure the linear transmission spectrum of the devices using an optical fiber coupling system. During the whole measurement, a super continuous laser (YSL SC-5) was used to excite the signal light from the single-mode fiber. The intensity of the signal light is adjusted by adjusting three polarizers, and then the signal light is focused on the coupling fiber. The optical fibers are placed on a precise three-dimensional displacement table. We optimize the near-field coupling with the grating coupler by carefully adjusting the position and angle of the incident fiber. Another single-mode fiber is symmetrically placed at the output coupling end of the grating coupler to collect the output signal light through near-field coupling. We normalized transmission by using reference samples with the same grating coupler parameters but no nano-structures.

The parameters of the super continuous laser are: maximum output power of is 800 mW, peak output power output from the optical fiber is 100 μW, central wavelength is 1064 nm, repetition frequency is 5MHz, the pulse width is 400 ps and the power ratio of 1550nm is 0.001. The coupling efficiency of the grating is 10%. Therefore, the energy of 1550 nm signal light and control light is calculated as 2 fJ /bit, that is kW/cm^2^. Compared to the energy consumption of electronic calculations, MW/cm^2^, it improved three orders of magnitude.
